# Chorioamnionitis appears not to be a Risk Factor for Patent Ductus Arteriosus in Preterm Infants: A Systematic Review and Meta-Analysis

**DOI:** 10.1038/srep37967

**Published:** 2016-11-28

**Authors:** Elham Behbodi, Eduardo Villamor-Martínez, Pieter L. J. Degraeuwe, Eduardo Villamor

**Affiliations:** 1 Department of Pediatrics, Maastricht University Medical Center (MUMC+), School for Oncology and Developmental Biology (GROW), Maastricht, the Netherlands

## Abstract

The contribution of chorioamnionitis (CA) to mortality and morbidity in preterm infants is difficult to assess because observational studies frequently present significant differences in baseline characteristics of the infants exposed or non-exposed to CA. In an attempt to perform a thorough assessment of the possible association between CA and patent ductus arteriosus (PDA) in preterm infants, we conducted a meta-analysis in which *adjusted* odds ratios (ORs) were pooled and we analyzed the effects of potential confounders, such as gestational age (GA) or birth weight (BW). We identified 45 relevant studies (27186 patients, 7742 CA cases). Random effects meta-analysis of crude ORs showed a significant positive association between CA and PDA (OR 1.352, 95% CI 1.172 to 1.560). Adjusted ORs were reported in 11 studies (19577 infants). Meta-analysis of these studies showed a significant negative association between CA and PDA (OR 0.802, 95% CI 0.751 to 0.959). Meta-regression showed that the differences in GA or BW between the CA-exposed and non-exposed groups were significantly correlated with the effect size of the association between PDA and CA. In conclusion, our study confirms that confounders need to be taken into account when assessing the association between CA and clinical outcomes in preterm infants.

The term chorioamnionitis (CA) refers to an intrauterine status of infection/inflammation in tissues of either mixed fetal-maternal (choriodecidual space) or fetal origin (chorioamniotic membranes, amniotic fluid, umbilical cord)^1^,^2^,^3^,^4^. CA is considered to be one of the main causes of preterm labor and its incidence increases with decreasing gestational age (GA). Moreover, CA may induce a fetal inflammatory response which is thought to play an important role in short- and long-term morbidity after very preterm birth^1^,^2^,^3^,^4^,^5^,^6^,^7^,^8^,^9^,^10^,^11^,^12^,^13^. In recent years, numerous observational studies have been summarized in several meta-analyses attempting to clarify the association between CA and neonatal brain injury^11^, cerebral palsy^6^, bronchopulmonary dysplasia (BPD)^7^,^10^, necrotizing enterocolitis (NEC)^12^, and retinopathy of prematurity (ROP)^13^, among other adverse outcomes of prematurity. Nevertheless, since CA is a major risk factor for spontaneous preterm birth, the GA-independent contribution of CA to mortality and morbidity of preterm infants is very difficult to assess^3^.

Patent ductus arteriosus (PDA) is a common clinical problem among very preterm infants^14^,^15^. Very recently, Park *et al*. conducted a meta-analysis aiming to investigate the possible association between CA and PDA^16^. This meta-analysis was based on 23 studies (17.708 preterm infants, 4681 CA events) and showed a significant association between CA and PDA with an odds ratio (OR) of 1.43 and a 95% confidence interval (CI) of 1.19 to 1.72^16^. However, an important limitation of the study was that confounding factors, such as GA, were not taken into account. Noteworthy is that the three largest cohort studies reporting on the association between CA and PDA, showed a significant positive crude OR but a significant negative association when the OR was adjusted for confounding factors^17^,^18^,^19^. Unfortunately, this relevant finding was missed in the meta-analysis of Park *et al*.^16^.

According to McElrath *et al*., the pregnancy disorders that lead to very preterm delivery can be divided into two broad groups^20^. One group is characterized by the presence of signs of infection/inflammation, but absence of indicators of impaired placentation. This group is associated with preterm labor, premature rupture of membranes (PROM), placental abruption, and cervical insufficiency. The second group is characterized by the relative absence of inflammation, but presence of histologic features of dysfunctional placentation. This group is associated with preeclampsia and the entity identified as fetal indication/intrauterine growth restriction. Therefore, observational studies comparing the outcomes of infants with and without CA are, in fact, comparing the effects of placental infection/inflammation with vascular placental pathology^10^,^21^. This may result in significant differences between the CA and the “control” group in terms of, for example, GA, birth weight (BW), or use of antenatal corticosteroids^10^,^20^,^21^,^22^. These differences may exert an important influence in outcomes such as PDA.

In an attempt to perform a more thorough assessment of the possible association between CA and PDA in preterm infants, we conducted a systematic review and meta-analysis in which adjusted ORs, whenever available, were pooled. In addition, we analyzed the magnitude of the differences in potential confounders, such as GA or BW, between the infants of the CA and the control group. Finally, we performed a meta-regression in order to investigate the effect of confounders on the association between CA and PDA.

## Results

### Description of studies

We identified 1188 potentially relevant studies from which 45 (27186 patients, 7742 CA cases, 8033 PDA cases) met the inclusion criteria (Supplementary Figure 1). The main characteristics of the included studies are shown in Supplementary Table 1. While all studies provided data to measure the association between CA and PDA, none of the studies was primarily designed to assess this association. In 40 studies^7^,^8^,^9^,^17^,^18^,^19^,^23^,^24^,^25^,^26^,^27^,^28^,^29^,^30^,^31^,^32^,^33^,^34^,^35^,^36^,^37^,^38^,^39^,^40^,^41^,^42^,^43^,^44^,^45^,^46^,^47^,^48^,^49^,^50^,^51^,^52^,^53^,^54^,^55^,^56^, the objective was to examine the outcomes, including PDA, of preterm infants with and without maternal CA. In 5 studies^57^,^58^,^59^,^60^,^61^, the objective was to examine the risk factors for PDA, including maternal CA. Ten studies^17^,^19^,^23^,^24^,^25^,^57^,^58^,^59^,^60^,^61^ dealt with clinical and 34^7^,^8^,^9^,^18^,^26^,^27^,^28^,^29^,^30^,^31^,^32^,^33^,^34^,^35^,^36^,^37^,^38^,^39^,^40^,^41^,^42^,^43^,^44^,^45^,^46^,^47^,^48^,^49^,^50^,^51^,^52^,^53^,^54^,^55^ dealt with histological CA. One study^56^ described intra-amniotic infection/inflammation. This study was pooled in the group of histological CA. The quality of each study according to the Newcastle-Ottawa Scale is summarized in the Supplementary Table 1. All studies included in the meta-analysis achieved at least six stars, indicating good quality.

#### Analysis based on unadjusted data

**Figure 1:**
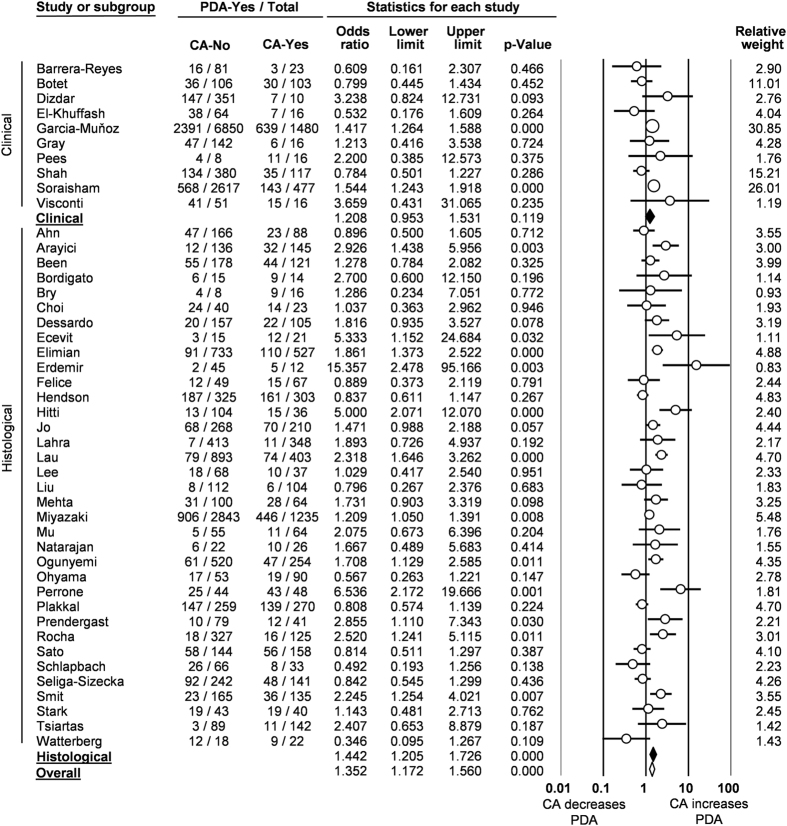
Forest plot for association between chorioamnionitis (CA) and patent ductus arteriosus (PDA). Unadjusted results.

The pooled unadjusted OR from the 45 studies showed a significant positive association between CA exposure and PDA (OR 1.352, 95% CI 1.172 to 1.560; Heterogeneity: Q = 123,1, P < 0.001, I^2^ = 64.3) (Fig. 1). The association remained significant for histological CA (OR 1.442, 95% CI 1.205 to 1.726; Heterogeneity: Q = 105.0, P < 0.001, I^2^ = 67.6) but not for clinical CA (OR 1.208, 95% CI 0.953 to 1.531; Heterogeneity: Q = 17.6, P = 0.040, I^2^ = 48.9) (Fig. 1). Neither visual inspection of the funnel plot (Supplementary Figure 2) nor the regression test of Egger (P = 0.517) revealed evidence of publication bias.

In order to explore the possible differences in baseline characteristics between the groups of exposed and non-exposed to CA, we performed a number of additional meta-analyses. As summarized in Table 1, infants exposed to CA showed significantly lower GA (Supplementary Figure 3) and BW (Supplementary Figure 4), significantly higher rates of exposure to antenatal corticosteroids, significantly higher rates of premature rupture of membranes (PROM), significantly lower rates of cesarean delivery, and significantly lower rates of preeclampsia.

**Figure 2:**
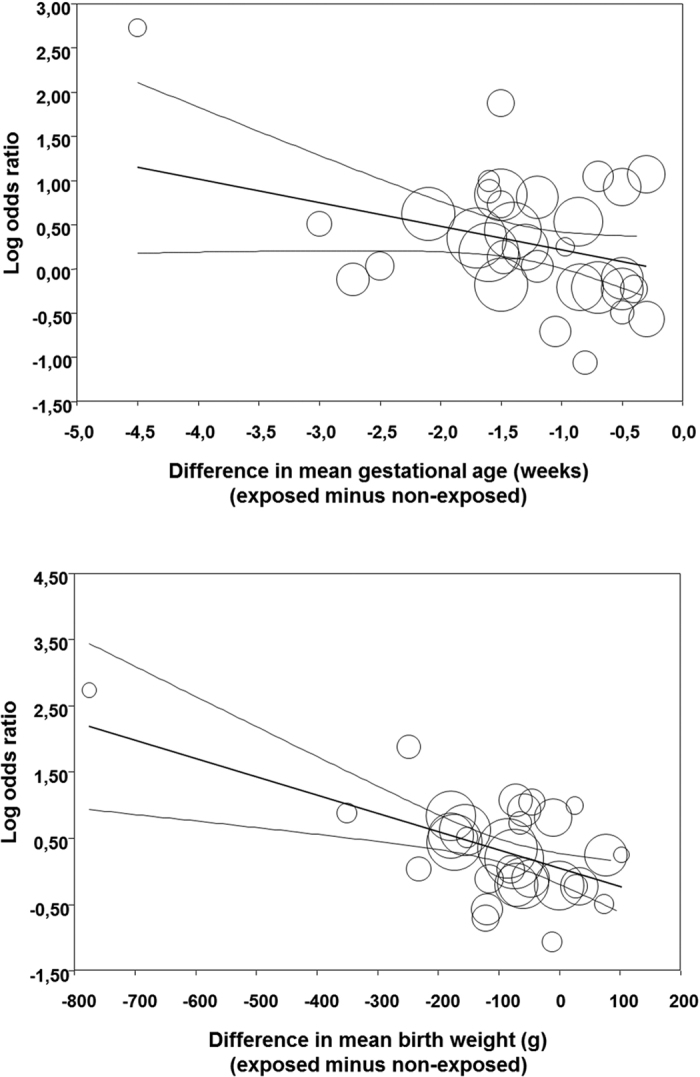
Meta-regression plot of association between chorioamnionitis and PDA controlling for difference in gestational age and birth weight between exposed and non-exposed groups.

In order to analyze the possible influence of the above mentioned baseline characteristics on the unadjusted association between CA and PDA, we performed a meta-regression analysis. As depicted in Table 2 and Fig. 2, this analysis showed that the differences in GA or BW between the CA exposed and non-exposed groups were significantly correlated with the effect size of the association between PDA and CA. In contrast, meta-regression could not demonstrate a significant effect of the rate of use of antenatal corticosteroids, mode of delivery, rate of SGA, rate of PROM, or rate of preeclampsia on the effect size of the different studies (Table 2).

Finally, we performed an additional analysis aimed at evaluating the role of the presence of a fetal inflammatory response (i.e., funisitis) on the development of PDA. Eight studies^8^,^9^,^26^,^27^,^33^,^35^,^40^,^41^,^46^ reported on PDA in infants with histological CA with or without funisitis. The pooled OR for PDA of the group with CA and funisitis (1.613, 95% CI 0.935 to 2.786 P = 0.086) was not significantly different (meta-regression coefficient: 0.233, 95% CI -0.405 to 0.817, P = 0.473) from the pooled OR of the group with CA without funisitis (1.322, 95% CI 0.975 to 1.792, P = 0.073) (Supplementary Figure 5).

#### Analysis based on adjusted data

**Figure 3:**
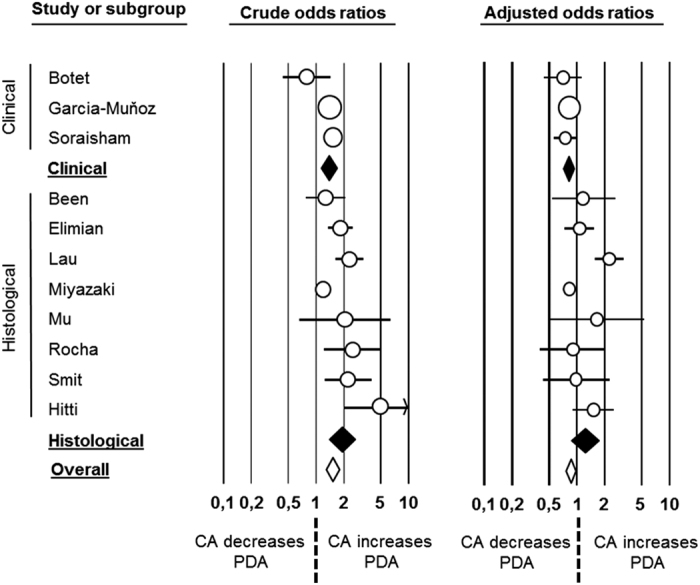
Forest plot for association between chorioamnionitis (CA) and patent ductus arteriosus (PDA). Unadjusted (left) and adjusted (right) results.

Adjusted ORs were reported in 8 studies^17^,^18^,^19^,^31^,^37^,^40^,^44^,^56^. Data on 3 additional studies^8^,^24^,^27^ were obtained from the authors. Therefore, a total of 11 studies (19577 infants) were included in these analysis that showed a significant negative association between CA and PDA (OR 0.802, 95% CI 0.751 to 0.959; Heterogeneity: Q = 35.0, P < 0.001, I^2^ = 71.4). This association remained significant for clinical (OR 0.849, 95% CI 0.703 to 0.916; Heterogeneity: Q = 0.7, P = 0.709, I^2^ = 0.0) but not for histological CA (OR 1.214, 95% CI 0.781 to 1.692; Heterogeneity: Q = 26.6, P < 0.001, I^2^ = 73.7) (Fig. 3, Table 3).

To investigate the effect of adjustment, the crude data of the above mentioned 11 studies were pooled and compared with the pooled adjusted ORs from the same studies (Fig. 3 and Table 3). The meta-analysis of this subgroup of crude data showed a significant positive association between CA and PDA (OR 1.524, 95% CI 1.291 to 1.800). The association remained significant for clinical (OR 1.383, 95% CI 1.134 to 1.686) and histological CA (OR 1.925, 95% CI 1.416 to 2.616). Meta-regression showed that the pooled crude ORs were significantly different than the pooled adjusted ORs from the same studies (P < 0.000001, P = 0.043, and P < 0.0008 for clinical, histological and any type of CA respectively).

## Discussion

There is a substantial body of evidence supporting that CA is a major risk factor for spontaneous preterm birth but the independent contribution of CA to prematurity-associated mortality and morbidity is much more difficult to assess^3^. The present study confirms that confounders need to be taken into account when assessing the association between CA and clinical outcomes in preterm infants. The meta-analysis of unadjusted data showed a significant positive association between CA and PDA, similar to the one reported in the study of Park *et al*.^16^. In contrast, the meta-analysis of adjusted data showed a significant negative association between CA and PDA. Moreover, our analyses provide data on the magnitude of the differences in GA, BW, rate of SGA, use of antenatal corticosteroids, and mode of delivery between infants exposed and non-exposed to CA. Meta-regression showed that differences in GA and BW between infants exposed and unexposed to CA may account for the higher risk of PDA observed when unadjusted data were pooled.

We used an extensive search strategy, which included not only studies describing PDA as outcome after exposure to CA, but also studies that assessed CA as potential risk factor for PDA. Through this search strategy, we identified 23 studies (9478 patients) which were not included in the study of Park *et al*.^16^. However, the key methodological limitation of the meta-analysis of Park *et al*. is the exclusive exploitation of unadjusted ORs^16^. Whereas descriptive analyses can still be done with such unadjusted data, meaningful statistical inference can be problematic^62^. When patient-level data are not available, confounding in meta-analysis can be reduced by using adjusted odds and/or hazard ratios from each source study^62^,^63^,^64^. By choosing to favor unadjusted ORs, Park *et al*. sacrificed patient-level data adjustment performed with a comprehensive set of predictors, leading to an overestimation in the strength of association between CA and PDA.

Meta-analysis of observational studies presents challenging methodological issues involving different study designs (i.e., cohort and case-control), variation in the quality of studies in terms of assessment of exposure and outcomes, missing data, control for confounding, or choice of controls in the case-control studies^62^,^63^,^64^. As underlined by Hartling *et al*. in their meta-analysis on the association between CA and BPD, studies reporting pooled data on outcomes of preterm infants exposed or unexposed to CA should take into account that the “control” group likely included infants with different baseline characteristics than the CA-exposed group^10^,^21^. As mentioned in the introduction, the pathophysiological processes that lead to very preterm delivery have been divided into two main categories^20^: intrauterine infection/inflammation and placental vascular dysfunction. In addition to distinct pathophysiological pathways, conditions of delivery are different between the two groups. In the vascular disease group, there is a higher incidence of caesarean section, growth restriction, and older GAs than in the infection/inflammation group^20^,^22^. Accordingly, our analyses showed that the infants exposed to CA were born significantly earlier (~1.3 weeks), were lighter (~75 g), presented growth restriction less frequently, had a higher rate of PROM, and had a lower rate of cesarean section. Meta-regression showed that the differences in GA and BW significantly influenced the association between CA and PDA. This is not surprising since both CA and PDA are inversely related to GA^1^,^2^,^15^,^65^,^66^ and highlights again the relevance of adequate correction, at least for this important confounder.

Previous meta-analyses on the relationship between CA and BPD^10^, cerebral palsy^6^, or ROP^13^ showed that the positive association observed with unadjusted data was significantly reduced, or became non-significant, when adjusted data were pooled. The differences that we observed herein are even more marked since the significant positive association between CA and PDA became a significant negative association when only adjusted data were taken into consideration. This suggests that CA may even exert a protective effect on the occurrence of PDA. A possible protective effect of CA in outcomes such as IRDS, or BPD has been reported in several individual studies and it has been suggested that CA exposure may protect the infants by promoting lung maturation and reducing the need for surfactant and mechanical ventilation^7^,^18^,^21^,^22^,^41^,^67^,^68^. On the other hand, the possible beneficial effect of CA might be erased by the frequent occurrence of postnatal pro-inflammatory events and complications such as sepsis^21^,^67^,^68^. Nonetheless, the relationship between PDA and respiratory condition in preterm infants is complex and bidirectional. In many instances, the presence of a large ductal shunt is suspected only on the basis of respiratory findings, such as increasing requirements for supplemental oxygen, or inability to reduce mechanical ventilator support^15^. Conversely, stimuli that alter pulmonary precapillary tone, such as surfactant administration or mechanical ventilation can alter the left-to-right PDA shunt^15^. Therefore, the possible effect of CA on PDA development might be mediated through the effects of CA on the clinical respiratory condition of the infants.

That the fetal inflammatory response induced by CA might specifically influence the closure of the ductus arteriosus (DA) is a biologically plausible hypothesis. In fact, neonatal sepsis is recognized as an important risk factor for developing a hemodynamically significant PDA. As reviewed by Vucovich *et al*.^66^, the possible mechanisms linking neonatal inflammation/infection and PDA include (i) hypoxia-induced DA relaxation due to respiratory insufficiency secondary to the inflammatory process; (ii) ductal relaxation mediated by components of bacteria, cytokines, or endogenous vasoactive mediators, such as prostaglandins, NO or CO; (iii) increased fluid administration in order to treat the increasing third space volume that often accompanies the inflammatory response; and (iv) administration of drugs such as aminoglycosides that are known relaxants of the DA^66^,^69^. Nevertheless, it should be considered that not all intraamniotic infections will lead to an inflammatory process extending to the fetal component^4^. Funisitis is considered the histologic counterpart of the fetal inflammatory response syndrome^4^. The present analysis showed that the presence of funisitis combined with CA did not significantly change the odds of having PDA, when compared with CA in the absence of funisitis. This is an argument against the fetal inflammatory response as etiopathogenic factor for PDA.

Maternal administration of corticosteroids in case of anticipated preterm delivery reduces neonatal mortality and morbidity and has become standard of care in current obstetric practice^70^,^71^. However, several concerns exist, or have existed, regarding the administration of antenatal steroids in cases of suspected intrauterine infection. Given their immunosuppressive effects, corticosteroids could theoretically activate or worsen infections and, therefore, some guidelines delineate CA as a contraindication for antenatal steroids^70^,^71^. Surprisingly, our meta-analysis shows that the rate of use of antenatal corticosteroids is higher in preterm infants exposed to CA when compared with the non-exposed infants (Table 2). Nevertheless, the higher rate of exposure to corticosteroids was only significant for the group of histological CA. Thus, it can be assumed that when the decision of starting corticosteroids was taken, clinicians did not suspect the presence of CA, at least in a number of patients. Of note is that two meta-analyses showed that administration of antenatal corticosteroids in patients with histological CA was linked to a significant reduction in PDA as well as in in mortality, RDS, and IVH^8^,^72^. In the present study, meta-regression could not demonstrate a significant influence of the rate of use of antenatal corticosteroids on the association between CA and PDA. However, the higher use of antenatal steroids in CA-exposed infants should be taken into account in future meta-analyses investigating the relationship between CA and neonatal outcomes.

Limitations of the literature and of our systematic review and meta-analysis deserve comment. First, the published literature showed great heterogeneity in definition of exposure, outcome, and in assessment of confounders. Second, we found no studies having the evaluation of the association between CA and PDA as main objective. Third, adjusted data were available only from 11 of the 45 studies included in the meta-analysis However, it should be noted that these 11 studies accounted for 72% of the infants and they were the studies with the highest quality. Nevertheless, we had to rely on the adjusted analyses as presented in the published reports and the variables which they included, which were not consistent across studies. On the other hand, the main strength of the present study is the use of rigorous methods including extensive and comprehensive search; duplicate screening, inclusion, and data extraction to reduce bias; and meta-regression to control for potential confounders.

In conclusion, the current meta-analysis demonstrates that the previously reported increased risk of PDA among preterm infants exposed to CA^16^, depends more on CA as etiological factor for preterm birth than on the possible effects of infection/inflammation on DA pathobiology. Our present results underscore the need for including all potential confounding factors in future observational studies on the outcomes of CA and performing analyses that adjust for these confounders and the possible interactions among them.

## Methods

The study was conducted according to the MOOSE guidelines for systematic review and meta-analysis of observational studies^73^. A protocol was developed prospectively that detailed the specific objectives, criteria for study selection, the approach to assessing study quality, clinical outcomes, and statistical methodology.

### Sources and search strategy

A comprehensive literature search was undertaken using the PubMed/MEDLINE and EMBASE databases from their inception to December 1, 2015. The search terms involved various combinations of the following keywords: “chorioamnionitis”, “intrauterine infection” “intrauterine inflammation” “prenatal infection” “prenatal inflammation”, “antenatal infection” “antenatal inflammation” “ductus arteriosus” “patent ductus arteriosus”, “risk factors”, “outcome”, “cohort”, and “case-control”. No language limit was applied. We performed additional searches by screening reference lists from articles of interest as well as citations to articles of interest, using the ISI Web of Knowledge and Google Scholar. We also contacted topic specialists to identify additional potentially relevant studies.

### Study selection

Studies were included if they had a CA and a comparison group, examined preterm or low BW infants, and reported primary data that could be used to measure the association between exposure to CA and the presence of a PDA. To identify relevant studies, two reviewers (EB, EV) independently screened the results of the searches and applied inclusion criteria using a structured form. Discrepancies were resolved through discussion or in consultation with a third reviewer (PD).

### Data extraction

Two investigators (EB, PD) independently extracted data from relevant studies using a predetermined data extraction form and another two investigators (EV-M, EV) checked data extraction for accuracy and completeness. Discrepancies were resolved by consulting the primary report. Data extracted from each study included citation information, language of publication, country where research was conducted, objectives, study design, definitions of CA and PDA, inclusion/exclusion criteria, patient characteristics, and results (including raw numbers and adjusted analyses on CA and PDA where available).

### Quality assessment

Methodological quality was assessed using the Newcastle-Ottawa Scale for cohort or case-control studies^74^. This scale uses a star rating system (range: 0–9 stars) scoring three aspects of the study: selection (0–4), comparability (0–2) and exposure/outcome (0–3). Two reviewers (PD and EV) independently assessed the methodological quality of each study. Discrepancies were resolved through discussion.

### Statistical Analysis

Studies were combined and analyzed using comprehensive meta-analysis V 3.0 software (Biostat Inc., Englewood, NJ, USA). For dichotomous outcomes, the OR with 95% CI was calculated from the data provided in the studies. ORs adjusted for potential confounders were extracted from the studies reporting these data. For continuous outcomes, the mean difference (MD) with 95% CI was calculated. When studies reported continuous variables as median and range or interquartile range, we estimated the mean and standard deviation using the method of Wan *et al*.^75^. Due to anticipated heterogeneity, summary statistics were calculated with a random-effects model. This model takes into account variability between studies as well as within studies. Subgroup analyses were conducted according to the mixed-effects model^76^. In this model a random-effects model is used to combine studies within each subgroup and a fixed-effect model is used to combine subgroups and yield the overall effect. The study-to-study variance (tau-squared) is not assumed to be the same for all subgroups. This value is computed within subgroups and not pooled across subgroups. Statistical heterogeneity was assessed by Cochran’s *Q* statistic and by the *I*^*2*^ statistic, which is derived from *Q* and describes the proportion of total variation that is due to heterogeneity beyond chance^77^. We used the Egger’s regression test and funnel plots to assess publication bias. To explore differences between studies that might be expected to influence the effect size, we performed univariate random-effects meta-regression (method of moments)^78^. The potential sources of variability defined a priori were: CA type (clinical or histological), differences in GA and BW between the infants with and without CA, use of antenatal corticosteroids, mode of delivery, rate of SGA, rate of PROM, and rate of preeclampsia. A probability value of less than 0.05 (0.10 for heterogeneity) was considered statistically significant.

## Additional Information

**How to cite this article**: Behbodi, E. *et al*. Chorioamnionitis appears not to be a Risk Factor for Patent Ductus Arteriosus in Preterm Infants: A Systematic Review and Meta-Analysis. *Sci. Rep.* **6**, 37967; doi: 10.1038/srep37967 (2016).

**Publisher's note:** Springer Nature remains neutral with regard to jurisdictional claims in published maps and institutional affiliations.

## Supplementary Material

Supplementary Figures and Table

## Figures and Tables

**Table 1:** Random effects meta-analyses of potential confounders.

Meta-analysis	Chorioamnionitis	k	Effect size	95% CI	Z	P	Heterogeneity		
							Q	P	I^2^
Gestational age (weeks)	Clinical	4	MD -1.151	−1.612 to −0.689	−4.888	<0.001	25.308	<0.001	88.146
	Histological	29	MD -1.418	−1.725 to −1.112	−9.070	<0.001	333.271	<0.001	91.958
	Any type	33	MD -1.336	−1.592 to −1.081	−10.260	<0.001	369.827	<0.001	91.347
Birth weight (g)	Clinical	4	MD -48	−130 to 34	−1.145	0.252	37.199	<0.001	91.935
	Histological	28	MD -80	−113 to −46	−4.659	<0.001	107.816	<0.001	74.957
	Any type	32	MD -75	−106 to −44	−4.745	<0.001	147.878	<0.001	79.037
Antenatal corticosteroids	Clinical	3	OR 1.498	1.024 to 2.191	−1.504	0.133	11.743	0.003	82.969
	Histological	26	OR 1.234	1.049 to 1.451	−4.549	<0.001	60.735	<0.001	58.837
	Any type	29	OR 1.271	1.095 to 1.475	3.156	0.002	72.503	<0.001	61.381
Cesarean section	Clinical	3	OR 0.434	0.389 to 0.485	−14.725	<0.001	1.695	0.428	0.000
	Histological	17	OR 0.373	0.297 to 0.469	−8.483	<0.001	83.466	<0.001	80.830
	Any type	20	OR 0.422	0.382 to 0.466	−16.953	<0.001	85.911	<0.001	77.884
PROM	Any type	16	OR 2.884	2.085 to 3.989	6.401	<0.001	107.419	<0.001	86.036
SGA	Any type	11	OR 0.341	0.211 to 0.549	4.423	<0.001	58.549	<0.001	82.920
Preeclampsia	Any type	6	OR 0.143	0.084 to 0.243	−7.182	<0.001	13.948	0.016	64.153

K: number of studies; PDA: patent ductus arteriosus; MD: mean difference (chorioamnionitis-exposed minus unexposed); OR: odds ratio (OR > 1 means increased risk in infants exposed to chorioamnionitis);; PROM: premature rupture of membranes; SGA: small for gestational age.

**Table 2:** Random effects meta-regression.

Meta-regression	k	Coefficient	95% CI	Z	P
Diff. mean gestational age (per week)	32	−0.266	−0.501 to −0.032	−2.22	0.026
Diff. mean gestational age (significant yes/no)	32	0.584	0.190 to 0.977	2.91	0.004
Diff. mean birth weight (per 100 g)	31	−0.277	−0.421 to −0.132	−3.75	0.000
Diff. mean birth weight (significant yes/no)	31	0.123	−0.208 to 0.455	0.73	0.466
Chorioamnionitis type (clinical/histological)	45	0.204	−0.161 to 0.569	1.10	0.273
Antenatal corticosteroids (log OR)	28	0.143	−0.194 to 0.480	0.83	0.406
Cesarean section (log OR)	21	0.083	−0.176 to 0.341	0.63	0.530
Early onset sepsis (log OR)	16	0.022	−0.124 to 0.168	0.29	0.770
late onset sepsis(log OR)	22	0.309	−0.135 to 0.752	1.36	0.173
Small for gestational age (log OR)	11	0.188	−0.266 to 0.643	0.81	0.416
Premature rupture of membranes (log OR)	16	−0.264	−0.579 to 0.051	−1.65	0.099

K = number of studies.

**Table 3:** Crude and adjusted ORs and confounders.

	Study or subgroup	Crude OR (95% CI)	P	Adjusted OR (95% CI)	P	Confounders included in analysis
Clinical	Botet	0.799 (0.445–1.434)	0.452	0.705 (0.443–1.122)	0.140	GA
	Garcia-Muñoz	1.417 (1.264–1.588)	0.000	0.830 (0.710–0.970)	0.019	GA, BW
	Soraisham	1.544 (1.243–1.918)	0.000	0.750 (0.561–1.002)	0.052	GA, BW, delivery mode, ACS, maternal hypertension, 5 min Apgar
	**Clinical**	1.383 (1.134–1.686)	0.001	0.802 (0.703–0.915)	0.001	
Histological	Been	1.278 (0.784–2.082)	0.325	1.172 (0.537–2.560)	0.691	GA, SGA, sex, multiple birth, delivery mode, preeclampsia, PROM, ACS
	Elimian	1.861(1.373–2.522)	0.000	1.060(0.740–1.519)	0.751	GA, BW, BW percentile, 5 min Apgar
	Lau	2.318 (1.646–3.262)	0.000	2.218(1.552–3.170)	0.000	GA, BW, delivery mode, multiple births, ACS, maternal hypertension, SGA, 5 min Apgar <7, SNAP-II score, NTISS score
	Miyazaki	1.209 (1.050–1.391)	0.008	0.830(0.698–0.987)	0.035	GA, BW, SGA, sex, maternal age, parity, diabetes, preeclampsia, PROM, NRFS, ACS, delivery mode
	Mu	2.075 (0.673–6.396)	0.204	1.653 (0.510–5.358)	0.402	GA
	Rocha	2.520(1.241–5.115)	0.011	0.900 (0.400–2.025)	0.799	GA, BW
	Smit	2.245 (1.254–4.021)	0.007	0.979 (0.428–2.237)	0.960	GA, SGA, sex, multiple birth, delivery mode, preeclampsia, PROM, ACS
	Hitti	5.000 (2.071–12.070)	0.000	1.500 (0.900–2.500)	0.120	BW
	**Histological**	1.925 (1.416–2.616)	0.000	1.214(0.871–1.692)	0.252	
	**Overall**	1.524 (1.29–1.80)	0.000	0.849 (0.751–0.959)	0.009	

GA: Gestational age, BW: Birth weight, ACS: Antenatal corticosteroids, SGA: Small for GA, PROM: Premature rupture of membranes SNAP-II: Score for Neonatal Acute Physiology, PROM: Premature rupture of membranes, NTISS: Neonatal therapeutic intervention scoring system, NRFS: Non-reassuring fetal status.
