# Supplementary material for: Chorioamnionitis appears not to be a Risk Factor for Patent Ductus Arteriosus in Preterm Infants: A Systematic Review and Meta-Analysis
Source: Sci Rep. 2016 Nov 28;6:37967. doi: 10.1038/srep37967 (PMC5125028; doi:10.1038/srep37967)
Supplement: Supplementary Figures and Table [file srep37967-s1.pdf]

Supplementary information

Chorioamnionitis appears not to be a Risk Factor for Patent Ductus Arteriosus in Preterm Infants: A Systematic Review and Meta-Analysis

Elham Behbodi, Eduardo Villamor-Martínez, Pieter L. J. Degraeuwe, Eduardo Villamor*

Department of Pediatrics, Maastricht University Medical Center (MUMC+), School for Oncology and Developmental Biology (GROW), Maastricht, the Netherlands.

*Correspondence to E.Villamor@mumc.nl

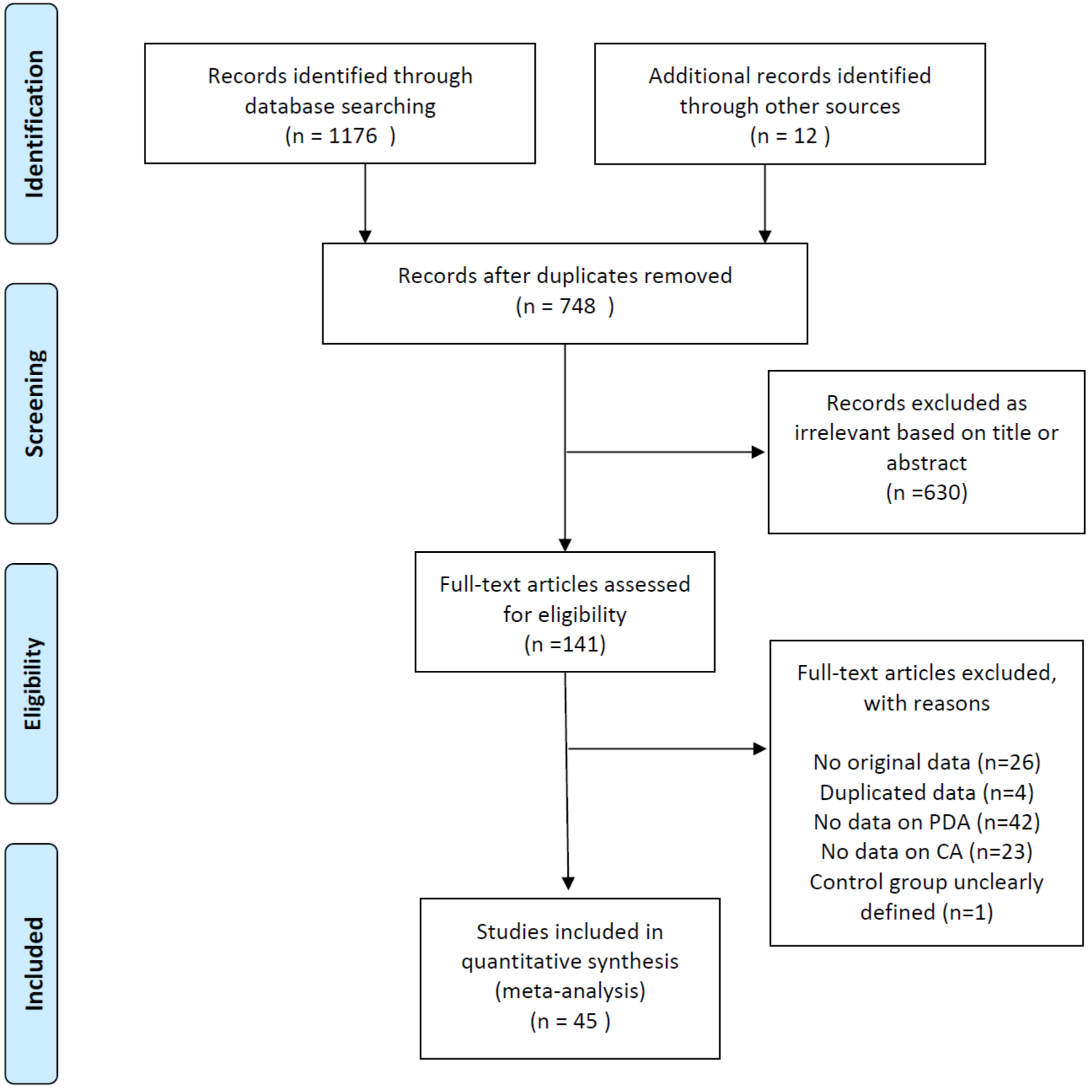

Supplementary Figure 1. Flow diagram of search results and study selection

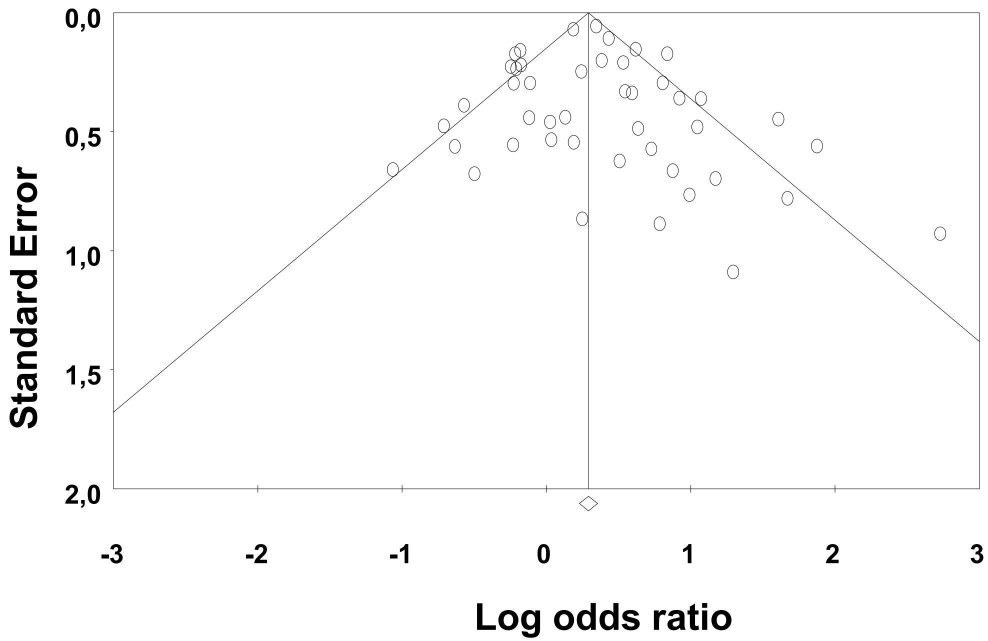

Supplementary Figure 2. Funnel plot for publication bias.
Egger's regresion intercept : 0.244 (95% CI -0,508 to 0,996, P=0,517)

Subgroup	Study or subgroup	Difference in means	Lower limit	Upper limit	p-Value	Relative weight
Clinical	Barrera-Reyes	-0.500	-1.231	0.231	0.180	17.91
Clinical	Botet	-0.500	-1.125	0.125	0.117	20.38
Clinical	Garcia-Muňoz	-1.700	-1.829	-1.571	0.000	31.76
Clinical	Soraisham	-1.400	-1.639	-1.161	0.000	29.95
	**Clinical**	-1.151	-1.612	-0.689	0.000	
Histological	Ahn	-0.500	-1.129	0.129	0.119	3.59
Histological	Arayici	-0.300	-0.897	0.297	0.325	3.64
Histological	Been	-1.300	-1.722	-0.878	0.000	3.91
Histological	Bordigato	-1.600	-2.329	-0.871	0.000	3.41
Histological	Bry	-0.971	-2.100	0.157	0.092	2.69
Histological	Choi	-2.500	-3.911	-1.089	0.001	2.23
Histological	Elimian	-2.100	-2.447	-1.753	0.000	4.01
Histological	Erdemir	-4.500	-5.843	-3.157	0.000	2.33
Histological	Felice	-2.730	-3.642	-1.818	0.000	3.07
Histological	Hendson	-1.500	-1.716	-1.284	0.000	4.14
Histological	Hitti	-4.200	-4.676	-3.724	0.000	3.83
Histological	Lau	-1.500	-1.934	-1.066	0.000	3.89
Histological	Lee	-1.800	-2.947	-0.653	0.002	2.65
Histological	Liu	-0.404	-0.839	0.031	0.069	3.89
Histological	Miyazaki	-1.600	-1.783	-1.417	0.000	4.16
Histological	Mu	-1.800	-2.968	-0.632	0.003	2.62
Histological	Natarajan	-3.000	-4.165	-1.835	0.000	2.62
Histological	Ogunyemi	-1.460	-1.813	-1.107	0.000	4.00
Histological	Ohyama	-0.300	-1.047	0.447	0.431	3.38
Histological	Perrone	-1.500	-2.236	-0.764	0.000	3.40
Histological	Plakkal	-0.700	-0.965	-0.435	0.000	4.09
Histological	Prendergast	-0.700	-1.387	-0.013	0.046	3.48
Histological	Rocha	-0.500	-0.867	-0.133	0.008	3.98
Histological	Sato	-0.850	-1.171	-0.529	0.000	4.04
Histological	Schlapbach	0.025	-0.551	0.601	0.932	3.68
Histological	Smit	-1.200	-1.608	-0.792	0.000	3.93
Histological	Stark	-1.480	-2.417	-0.543	0.002	3.03
Histological	Tsiartas	-1.570	-2.493	-0.647	0.001	3.05
Histological	Waterberg	-0.808	-1.616	0.000	0.050	3.26
	**Histological**	-1.418	-1.725	-1.112	0.000	
	**Overall**	-1.336	-1.592	-1.081	0.000	

Statistics for each study

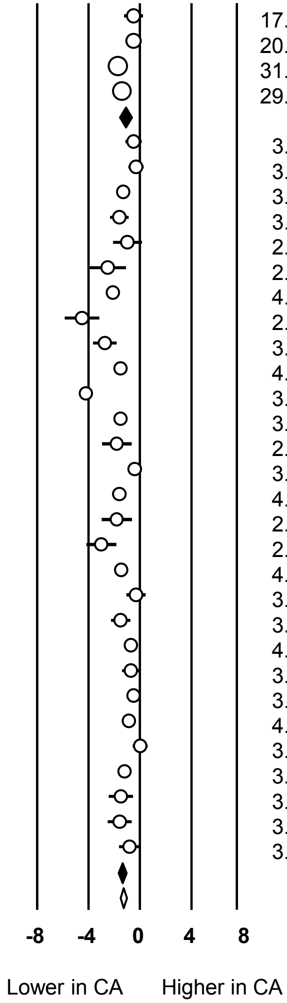

Supplementary Figure 3. Forest plot for difference in mean gestational age (weeks) of infants with and without exposure to chorioamnionitis (exposed minus unexposed).

Subgroup	Study or subgroup	Difference in means	Lower limit	Upper limit	p-Value	Relative weight
Clinical	Barrera-Reyes	74	-28	177	0.154	19.97
	Botet	33	-31	97	0.315	24.59
	Garcia-Muňoz	-85	-101	-70	0.000	28.73
	Soraisham	-173	-218	-128	0.000	26.71
		-48	-130	34	0.252	
Histological	Ahn	-48	-175	80	0.465	3.23
	Arayici	-72	-148	4	0.065	4.61
	Been	77	-3	157	0.060	4.49
	Bordigato	26	-103	155	0.694	3.18
	Bry	103	-24	230	0.113	3.24
	Choi	-232	-479	15	0.066	1.42
	Elimian	-155	-194	-116	0.000	5.58
	Erdemir	-775	-1086	-465	0.000	0.98
	Felice	-116	-226	-6	0.039	3.66
	Hendson	-60	-92	-28	0.000	5.73
	Hitti	-125	-346	96	0.267	1.67
	Lau	-179	-283	-75	0.001	3.82
	Lee	-80	-180	20	0.117	3.93
	Liu	27	-84	138	0.632	3.65
	Miyazaki	-74	-122	-26	0.003	5.38
	Mu	-64	-351	223	0.663	1.11
	Natarajan	-152	-532	228	0.433	0.69
	Ogunyemi	-179	-282	-76	0.001	3.85
	Ohyama	-119	-240	2	0.053	3.40
	Perrone	-248	-372	-124	0.000	3.32
	Plakkal	0	-39	39	1.000	5.60
	Prendergast	-45	-156	66	0.428	3.64
	Rocha	-58	-133	18	0.137	4.62
	Sato	-71	-130	-12	0.017	5.11
	Schlapbach	-121	-245	2	0.054	3.33
	Smit	-10	-91	71	0.808	4.49
	Tsiartas	-350	-556	-144	0.001	1.85
	Waterberg	-11	-94	72	0.793	4.41
		-80	-113	-46	0.000	
		-75	-106	-44	0.000	

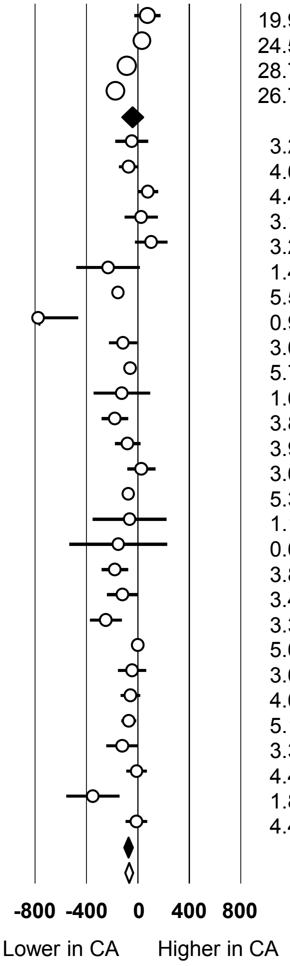

Supplementary Figure 4. Forest plot for difference in mean birth weight (grams) of infants with and without exposure to chorioamnionitis (exposed minus unexposed).

Funisitis negative

Study	Odds ratio	Lower limit	Upper limit	p-Value
Been	1,468	0,777	2,771	0,237
Dessardo	0,623	0,223	1,742	0,367
Lau	1,823	1,106	3,005	0,019
Liu	0,780	0,198	3,067	0,722
Ohyama	0,639	0,295	1,382	0,255
Plakkal	1,120	0,679	1,848	0,656
Smits	2,197	1,130	4,273	0,020
Tsiartas	1,890	0,458	7,795	0,379
Lahra	2,148	0,671	6,880	0,198
**Overall**	1,322	0,975	1,792	0,073

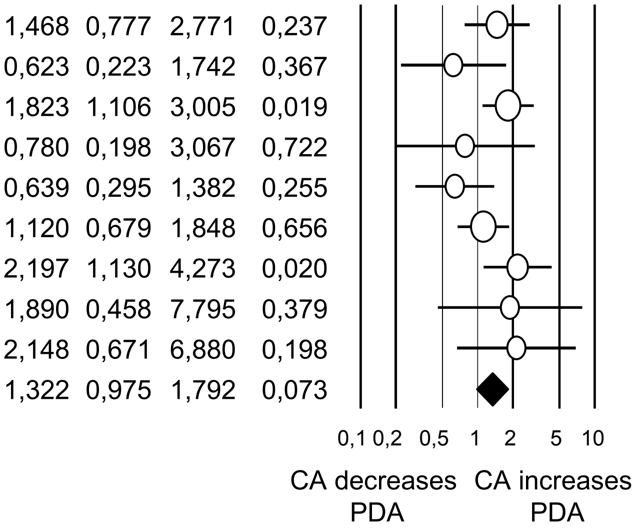

Funisitis positive

Study	Odds ratio	Lower limit	Upper limit	p-Value
Been	1,143	0,631	2,071	0,659
Dessardo	4,159	1,938	8,926	0,000
Lau	2,641	1,797	3,879	0,000
Liu	0,813	0,206	3,198	0,766
Ohyama	0,123	0,007	2,249	0,157
Plakkal	0,699	0,479	1,021	0,064
Smits	2,315	1,106	4,848	0,026
Tsiartas	3,583	0,816	15,737	0,091
Lahra	1,723	0,572	5,193	0,334
**Overall**	1,613	0,935	2,786	0,086

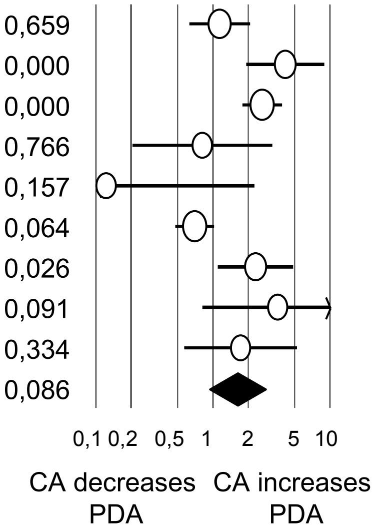

Supplementary Figure 5. Forest plot for association between chorioamnionitis (CA) without or with funisitis and patent ductus arteriosus (PDA).

Supplementary Table 1. Characteristics of the included studies.

First author, year	Place, Country	Study type	Prosp/ Retro	Total infants (centers)	Mean BW (g)	Mean GA (weeks)	Male (%)	ACS (%)	CA category	Incidence of CA (%)	Definition of CA	Incidence of PDA (%)	Definition of PDA	NOS score
Ahn, 2012	Seoul, Korea	Cohort (CA)	Prosp	254 (1)	1536	30.6	60	58	HC	35	Ref	28	Echo2	8
Arayici, 2014	Ankara, Turkey	Ca-Co (CA),	Retro	281 (1)	1173	28.9	55	71	HC	52	Des	16	Echo1	7
Barrera-Reyes, 2011	Mexico, Mexico	Cohort (CA	Prosp	104 (1)	1071	30.0	52		CC	22	Ref	18	NA	7
Been, 2009	Rotterdam, the Netherlands	Cohort (CA	Prosp	299 (1)	1143	29.1	52	71	HCF	40	Ref	33	Treat	8
Bordigato, 2011	Padova, Italy	Cohort (CA)	?	29 (1)	805	26.7	59	76	HC	48	Ref	52	NA	6
Botet, 2010	Spain	Ca-Co (CA)	Prosp	209 (12)	1056	28.3	53	89	CC	49	Des	32	NA	7
Bry, 2015	Gothenburg, Sweden	Cohort (CA)	Retro	24 (1)	777	25.9	50	100	HC	67	Ref	54	NA	6
Choi, 2008	Seoul, Korea	Cohort (CA)	Prosp	63 (2)	1243	29.2	49	59	HC	37	Ref	60	Echo2	8
Dessardo, 2014	Rijeka, Croatia	Cohort (CA)	Prosp	262 (1)	1317	29.2	49		HC	40	Ref	16	Echo2	8
Dizdar, 2012	Ankara, Turkey	Ca-Co (PDA)	Retro	361 (1)	1060	28.0	47		CC	3	NoDes	43	Echo1	6
Ecevit, 2014	Ankara, Turkey	Cohort (CA)	Retro	36 (1)	1523	29.5			HC	58	Ref	42	NA	6
Elimian, 2000	Valhalla, USA	Cohort (CA)	?	1260 (1)	1183	29.0		33	HC	42	Ref	16	Treat	7
El-Khuffash, 2008	Dublin, Ireland	Cohort (PDA)	Prosp	80 (1)	1066	28.5	56		CC	20	NoDes	56	Echo1	6
Erdemir, 2013	Izmir, Turkey	Cohort (CA)	Prosp	57 (1)	1675	30.8	46	49	HC	21	Des	12	NA	7
De Felice, 2001	Seiena and Brindisi, Italy	Cohort (CA)	Prosp	116 (2)	977	28.1	48		HC	58	Ref	23	NA	7
Garcia-Munoz, 2014	Spanish Network	Cohort (CA)	Prosp	8330 (53)	1086	28.5	52	67	CC	18	Des	36	Treat	9
Gray, 1997	Brisbane, Queensland, Australia	Cohort (CA)	?	158 (1)					CC	10	Des	34	Clin	6
Hendson, 2011	Edmonton, Canada	Cohort (CA)	Prosp	628 (1)	930	26.9	48	83	HC	48	Des	55	Echo2	8
Hitti, 2001	Seattle, USA	Cohort (CA)	Prosp	140 (3)	1657	30.8		51	HC[a]	26	Des	20	Echo1	8
Jo, 2004	Seoul, Korea	Cohort (CA)	Retro	478 (1)		30.7			HC	44	NoDes	29	NA	6

Supplementary Table 1 (cont.). Characteristics of the included studies.

First author, year	Place, Country	Study type	Prosp/ Retro	Total infants (centers)	Mean BW (g)	Mean GA (weeks)	Male (%)	ACS (%)	CA category	Incidence of CA (%)	Definition of CA	Incidence of PDA (%)	Definition of PDA	NOS score
Lahra, 2009	New South Wales, Australia	Cohort (CA)	Retro	761 (1)		27.4	52.4	60.6	HCF	45.7	Ref	2.2	Treat	8
Lau, 2005	Canadian Neonatal Network	Cohort (CA)	Prosp	1296 (17)	2068	33.2	55	47	HCF	31	Ref	12	Treat	9
Lee, 2006	Hong Kong	Cohort (CA)	Retro	105 (1)	1042	28.5	53	78	HC	35	Ref	27	Treat	7
Liu, 2014	Shangia, China	Cohort (CA)	Prosp	216 (2)	1705	31.7	58	41	HCF	48	Ref	6	Echo2	8
Mehta, 2006	New Brunswick, USA	Cohort, (CA)	Retro	164 (1)					HC	39	Ref	36	NA	6
Miyazaki, 2015	Neonatal Research Network, Japan	Cohort (CA)	Retro	4078 (66)	973	27.6	49	41	HC	30	Ref	33	Clin	8
Mu, 2008	Taipei, Taiwan	Cohort (CA)	Prosp	119 (1)	1108	28.6	54	45	HC	54	Ref	13	Echo2	7
Natarajan, 2008	Detroit, USA	Cohort (CA)	Prosp	48 (1)	975	27.4	52	46	HC	54	NoDes	31	Echo2	7
Ogunyemi, 2003	Los Angeles, USA	Cohort (CA)	Retro	774 (1)	1313	29.4		53	HC	33	Ref	14	NA	7
Ohyama, 2002	Yokohama, Japan	Cohort (CA)	Retro	143 (1)	1162	27.8			HCF	63	Ref	25	Echo2	8
Pees, 2010	Berlin, Germany	Cohort (PDA)	?	24 (1)	851	26.1	58		CC	67	NoDes	63	Echo1	7
Perrone, 2012	Siena, Italy	Cohort (CA)	Prosp	95 (1)	994	26.7			HC	54	Ref	72	Echo1	7
Plakkal, 2013	Calgery, Canada	Cohort (CA)	Retro	529 (1)	861	26.0	54	84	HCF	51	Des	54	Treat	7
Prendergast, 2011	London, UK	Cohort (CA)	Retro	120 (1)	1190	28.2	48	88	HC	34	Ref	18	Echo2	7
Rocha, 2006	Porto, Portugal	Cohort (CA)	Retro	452 (3)	1499	29.4		65	HC	28	Ref	8	Echo1	8
Sato, 2011	Yokohama, Japan	Cohort (CA)	Retro	302 (1)	904	26.3	52	61	HC	52	Ref	38	Treat	7
Schlapbach, 2010	Bern, Switzerland	Ca-Co (CA)	Retro	99 (1)	1244	28.8	49	83	HC	33	NoDes	34	NA	7

Supplementary Table 1 (cont.). Characteristics of the included studies.

First author, year	Place, Country	Study type	Prosp/ Retro	Total infants (centers)	Mean BW (g)	Mean GA (weeks)	Male (%)	ACS (%)	CA category	Incidence of CA (%)	Definition of CA	Incidence of PDA (%)	Definition of PDA	NOS score
Seliga-Sizecka, 2013	Warsaw, Poland	Cohort (CA)	Prosp	383 (1)	1338	29.2	56	48	HC	37	Ref	37	Echo1	7
Shah, 2011	San francisco, USA	Cohort (PDA)	Prosp	497 (1)	825	25.8	52		NA	24	NoDes	34	Echo1	7
Smit, 2015	Veldhoven, the Netherlands[b]	Cohort (CA)	Retro	300 (1)	1303	29.5	54	92	HCF	45	Ref	20	Treat	7
Soraisham, 2009	Canadian Neonatal Network	Cohort (CA)	Prosp	3094 (24)	1320	28.9	53	79	CC	15	Des	23	Treat	9
Stark, 2015	Adelaide, Australia	Cohort (CA)	Prosp	83 (1)		26.7	54	61	HC	48	Ref	46	Echo2	7
Tsiartas, 2013	Královec, Czech Republic	Cohort (CA)	?	231 (1)	1975	33.0		56	HC	61	Ref	6	NA	7
Visconti, 2013	São Paulo, Brasil	Cohort (PDA)	Retro	67 (1)	1063	28.2	63	72	NA	24	NoDes	84	Echo1	7
Waterberg, 1999	Pennsylvania, USA	Cohort (CA)	Prosp	40 (2)	751	25.3	38	85	HC	55	NoDes	53	Treat	7

ACS, antenatal corticosteroids; CA, chorioamnionitis; PDA, patent ductus arteriosus. NOS, Newcastle-Ottawa quality assessment scale. Abbreviations for study design: Ca-Co, case-control study; (CA), study analyzed PDA as outcome of chorioamnionitis; (PDA) study analyzed chorioamnionitis as risk factor for PDA; Prosp, prospective; Retro, retrospective; ?, unknown. Chorioamnionitis category: CC, clinical chorioamnionitis; HC, histological chorioamnionitis; HCF, histological chorioamnionitis with funisitis mentioned separately. Definitition of chorioamnionitis: NoDes, no description; Des, clinical or histological description; Ref, literature citation. Ascertainment of PDA: Echo1, echocardiography clearly defined; Echo2, echocardiography not clearly defined; Treat, medically or surgically treated PDA; NA, no diagnostic criteria mentioned.

[a]The study described intra-amniotic infection/inflammation but was pooled in the group of histological CA. [b]The study included a second group from Rotterdam that was also reported in the study of Been et al.
